# Correction: ‘I cannot be what I don’t see’: an evaluation of Academic Intersectionality Mentoring in medical schools (AIMMS Mentoring)

**DOI:** 10.1371/journal.pone.0335488

**Published:** 2025-10-27

**Authors:** The PLOS ONE Staff

## Notice of Republication

This article was republished on October 10, 2025, to correct an error in the XML and PDF. In the XML, the Author Roles were missing for Jane Illés. The Author Roles for Jane Illés should have been listed as follows: Conceptualization, Writing – review & editing. In the PDF, the fourth author’s name was spelled incorrectly in the Author Contributions section on pages 13 and 14. The correct name is: Jane Illés.

The publisher apologizes for these errors. The originally published, uncorrected article and the republished, corrected articles are provided here for reference.

## References

[1] Woodrow M, Benedikz E, Bryant LD, Illés J, Jagpal P, Jennings HM, et al. “I cannot be what I don’t see”: an evaluation of Academic Intersectionality Mentoring in medical schools (AIMMS Mentoring). PLoS One. 2025;20(4):e0318326. doi: 10.1371/journal.pone.0318326 40299878 PMC12040110

