# Supplementary material for: ‘I cannot be what I don’t see’: an evaluation of Academic Intersectionality Mentoring in medical schools (AIMMS Mentoring)
Source: PLoS One. 2025 Apr 29;20(4):e0318326. doi: 10.1371/journal.pone.0318326 (PMC12040110; doi:10.1371/journal.pone.0318326)
Supplement: S1 Supporting information — (PDF) [file pone.0318326.s001.pdf]

# Mentee or peer: baseline feedback form

Thank you for providing your feedback about the mentoring scheme in this short questionnaire. It should take you no more than 5 minutes to complete.

* Required

1. What is your current job title? (optional)

2. How would you describe your ethnicity? (optional)

3. What is your age? (optional)

- ◯ 18-24
- ◯ 25-34
- ◯ 35-44
- ◯ 45-54
- ◯ 55-64
- ◯ 65-74
- ◯ 75-84
- ◯ over 84

4. Do you feel that having a successful career in academic medicine has been more challenging because you are a woman from an ethnic minority? *

- ◯ Yes
- ◯ No

5. Please briefly explain your answer to Question 4 *

6. What do you want to get out of the mentoring scheme?  Please select all that apply *

- [ ] Professional development e.g. research skills, grant applications
- [ ] Personal development e.g. confidence, leadership
- [ ] To develop contacts and networks
- [ ] To enhance my CV and/or portfolio
- [ ] To receive practical advice about how to progress my career
- [ ] To support women from ethnic minorities in academic medicine
- [ ] General support
- [ ] Other

7. If you answered 'other' to Question 6, please expand if you would like

This content is neither created nor endorsed by Microsoft. The data you submit will be sent to the form owner.

Microsoft Forms

# Mentee or peer: follow up feedback form

Thank you for providing your feedback about the mentoring scheme by completing this short questionnaire. It should take you no more than 10min to complete.

* Required

1. What is your current job title?

2. How would you describe your ethnicity?

3. What age are you?

- ◯ 18-24
- ◯ 25-34
- ◯ 35-44
- ◯ 45-54
- ◯ 55-64
- ◯ 65-74
- ◯ 75-84
- ◯ over 84

4. How would you rate your experience of the mentoring scheme? *

☆ ☆ ☆ ☆ ☆

5. Did your experience of the scheme match what you expected? *

- ◯ Yes
- ◯ No

6. Please feel free to expand on your answer to Question 5

7. How many times have you and your mentor/peer met as part of the scheme? *

- ○ Not yet met
- ○ 1
- ○ 2
- ○ 3
- ○ 4
- ○ 5
- ○ More than five times

8. Are you currently still being mentored by your mentor? *

- ○ Yes
- ○ No

9. Please rate the following statements *

| | Strongly agree | Agree | Neutral | Disagree | Strongly disagree |
|---|---|---|---|---|---|
| I have a good relationship with my mentee/peer | ◯ | ◯ | ◯ | ◯ | ◯ |
| The support available from the scheme for me to undertake mentoring was helpful | ◯ | ◯ | ◯ | ◯ | ◯ |
| I felt supported by others to take part in the scheme e.g by my department, colleagues | ◯ | ◯ | ◯ | ◯ | ◯ |

10. What did you find most rewarding from taking part in mentoring? *

11. What did you find most challenging? *

12. How could the scheme be improved? *

13. Mentoring has helped me develop personally in the following areas (please select all that apply) *

- [ ] Interpersonal/communication skills
- [ ] Self confidence/validation
- [ ] Leadership
- [ ] Goal setting
- [ ] Collaborative working
- [ ] Dealing with conflict
- [ ] Negotiating
- [ ] Understanding of other perspectives
- [ ] Balancing competing needs
- [ ] Achieving a good work-life balance
- [ ] Developing independence
- [ ] Opportunity for self reflection
- [ ] I have not developed personally
- [ ] Other

14. Please feel free to expand about how you have developed personally

15. Mentoring has helped me develop professionally in the following areas (please select all that apply) *

- [ ] Expansion of professional networks
- [ ] Academic responsibilities, knowledge and skills
- [ ] Management and development skills
- [ ] Increased productivity
- [ ] Performance at job interviews
- [ ] CV development
- [ ] Insight into career development opportunities, promotion criteria, methods
- [ ] Insight into institutional ways of working
- [ ] Insight into the nature of academic life as a woman from an ethnic minority
- [ ] I have not developed professionally
- [ ] Other

16. Please feel free to expand about how you have developed professionally

17. Have you taken part in mentoring previously? *

- ☐ Yes as a mentor
- ☐ Yes as a mentee
- ☐ No

18. Having participated in this mentoring scheme, how confident are you that it will positively impact on you or your career? *

| 0 | 1 | 2 | 3 | 4 | 5 | 6 | 7 | 8 | 9 | 10 |
|---|---|---|---|---|---|---|---|---|---|---|

Not at all confident — Extremely confident

19. Having participated in the scheme, how useful is it that mentoring takes place between people with similar characteristics? *

| 0 | 1 | 2 | 3 | 4 | 5 | 6 | 7 | 8 | 9 | 10 |
|---|---|---|---|---|---|---|---|---|---|---|

Not at all useful — Very useful indeed

20. Please feel free to expand upon your answer to Question 19

21. Would you recommend this scheme to others? *

- Yes
- No

22. Please feel free to expand on your answer to Question 21

This content is neither created nor endorsed by Microsoft. The data you submit will be sent to the form owner.

Microsoft Forms

# Mentor: baseline feedback form

Thank you for providing your feedback about the mentoring scheme in this short questionnaire.
It should take you no more than 5 minutes to complete.

* Required

1. What is your current job title? (optional)

2. How would you describe your ethnicity? (optional)

3. What is your age? (optional)

- 18-24
- 25-34
- 35-44
- 45-54
- 55-64
- 65-74
- 75-84
- over 84

4. Do you feel that having a successful career in academic medicine has been more challenging because you are a woman from an ethnic minority? *

- Yes
- No

5. Please briefly explain your answer to Question 4 *

6. What would you like to get out of the mentoring scheme?  Please select all that apply *

- ☐ Professional development
- ☐ Personal development
- ☐ To support women from ethnic minorities in academic medicine
- ☐ To develop contacts and networks
- ☐ To increase my understanding about the difficulties facing women from ethnic minorities in academic medicine
- ☐ To enhance my CV and/or portfolio
- ☐ Other

7. If you answered 'other' to Question 6, please expand if you would like

This content is neither created nor endorsed by Microsoft. The data you submit will be sent to the form owner.

Microsoft Forms

# Mentor: follow up feedback form

Thank you for providing your feedback about the mentoring scheme in this short questionnaire. It should take you no more than 10 minutes to complete.

* Required

1. What is your current job title? (optional)

2. How would you describe your ethnicity? (optional)

3. What is your age? (optional)

- ○ 18-24
- ○ 25-34
- ○ 35-44
- ○ 45-54
- ○ 55-64
- ○ 65-74
- ○ 75-84
- ○ over 84

4. How would you rate your experience of the mentoring scheme? *

☆ ☆ ☆ ☆ ☆

5. Did you get out of the scheme what you expected? *

- ○ Yes
- ○ No

6. Please feel free to expand on your answer to Question 5

7. How many times have you and your mentee met as part of the scheme? *

- Not yet met
- 1
- 2
- 3
- 4
- 5
- More than five times

8. Are you currently still mentoring your mentee? *

- Yes
- No

9. Please rate the following statements *

| | Strongly agree | Agree | Neutral | Disagree | Strongly disagree |
|---|---|---|---|---|---|
| I have a good relationship with my mentee/peer | ○ | ○ | ○ | ○ | ○ |
| The support available from the scheme for me to undertake mentoring was helpful | ○ | ○ | ○ | ○ | ○ |
| I felt supported by others to take part in the scheme e.g. by my department, colleagues | ○ | ○ | ○ | ○ | ○ |

10. What did you find most rewarding from taking part in mentoring? *

11. What did you find most challenging? *

12. How could the scheme be improved? *

13. Mentoring has helped me develop personally in the following areas *

- [ ] Interpersonal/communication skills
- [ ] Listening skills
- [ ] Self confidence/validation
- [ ] Leadership
- [ ] Mentoring skills
- [ ] Goal setting
- [ ] Collaborative working
- [ ] Dealing with conflict
- [ ] Negotiating
- [ ] Understanding of other perspectives
- [ ] Achieving a good work-life balance
- [ ] Opportunity for self reflection
- [ ] I have not developed personally
- [ ] Other

14. Please feel free to expand about how you have developed personally

15. Mentoring has helped me develop professionally in the following areas *

- [ ] Management and development skills
- [ ] Expansion of professional networks
- [ ] Academic knowledge and skills
- [ ] Insight into institutional ways of working
- [ ] Insight into the nature of academic life as a woman from an ethnic minority
- [ ] CV development
- [ ] I have not developed professionally
- [ ] Other

16. Please feel free to expand about how you have developed professionally

17. Have you taken part in mentoring previously? *

- [ ] Yes as a mentor
- [ ] Yes as a mentee
- [ ] No

18. Having participated in this mentoring scheme, how confident are you that it will positively impact on you or your career? *

| 0 | 1 | 2 | 3 | 4 | 5 | 6 | 7 | 8 | 9 | 10 |
|---|---|---|---|---|---|---|---|---|---|---|

Not at all confident — Extremely confident

19. Having participated in the scheme, how useful is it that mentoring takes place between people with similar characteristics? *

| 0 | 1 | 2 | 3 | 4 | 5 | 6 | 7 | 8 | 9 | 10 |
|---|---|---|---|---|---|---|---|---|---|---|

Not at all useful — Very useful indeed

20. Please feel free to expand upon your answer to Question 19

21. Would your recommend this mentoring scheme to others? *

- ◯ Yes
- ◯ No

22. Please feel free to expand on your answer to Question 21

This content is neither created nor endorsed by Microsoft. The data you submit will be sent to the form owner.

Microsoft Forms
