# Supplementary material for: ‘I cannot be what I don’t see’: an evaluation of Academic Intersectionality Mentoring in medical schools (AIMMS Mentoring)
Source: PLoS One. 2025 Apr 29;20(4):e0318326. doi: 10.1371/journal.pone.0318326 (PMC12040110; doi:10.1371/journal.pone.0318326)
Supplement: Attachment — Submitted filename: Response to reviewers.docx [file pone.0318326.s003.docx]

**‘I cannot be what I don’t see’: an evaluation of Academic Intersectionality Mentoring in Medical Schools (AIMMS Mentoring)**

**Response to reviewers**

August 2024

We thank the reviewers for their time and expertise providing their reviews to improve our manuscript. Please find below a point-by-point response. All references to line numbers in our response below are references to the tracked change version of the manuscript. We have taken the opportunity to make very minor improvements and clarifications to the manuscript in addition to the amendments made in response to reviewers’ comments.

**Reviewer 1 comments**

This is a very interesting study that describes a mentoring scheme developed for women from ethnic minority backgrounds. The study is interesting, but there are numerous points of improvement for publication. First, more needs to be done to describe the AIMMS and the evaluation of the scheme. The initial development and implementation of the scheme needs more attention.

Thank you very much for your considered feedback. We hope we have been able to take account of all your comments in our revisions.

*Introduction*
Line 60 – typo “ither” should be “other”

Thank you, we have amended (line 63).

Line 69 – HE needs to be defined.
‘AdvanceHE’ is the full name of the organisation (line 91).

Line 71 – HEI needs to be defined

Thank you, we have expanded (line 93).

Line 84 – Clarification is needed about the area that has limited published research. Is it regarding mentoring as a solution for addressing inequalities in the workplace? Or mentoring in the workplace in general?

Thank you, we have edited this sentence to make it clearer (line 107).

Line 95 – NAA needs to be defined
Thank you, we have rephrased (line 121).

Line 107 – From July 2021 - ?
The committee continuously promotes the scheme, so “from July 2021” is superfluous and we have deleted this (line 133).

The introduction needs a stronger flow in the first 3 paragraphs. The concept of intersectionality doesn’t span beyond the single paragraph, it seems disconnected from the rest of the intro despite seeming important to the scheme.
We thank the reviewer for this suggestion to improve the introduction section. We have now restructured and added text to explain the importance of adopting an intersectional approach to addressing inequities and given some examples (lines 69-90). Minor amendments have also been made to the abstract.

The description of the AIMMS should be all together. Everything that describes the overall mentoring program should not be split between the introduction and methods.

Thank you, we have moved the description of the scheme to the introduction section (‘Scheme procedure’) and included a new section called ‘Recruitment and data collection’ within the Methods.

*Methods*
The scheme procedure section seems to bounce around between talking about how participants were recruited to be a part of the mentoring scheme and the evaluation.

Thank you. As explained above, we have restructured the Introduction and Methods to bring more clarity.

How did women become aware of the scheme? How/who did they contact if they were interested?

Due to word count we did not go into this level of detail in our first draft, but are delighted to provide further detail and clarity. We have included information about awareness in lines 156-160.

Is recruitment for the evaluation the same as recruitment for the scheme?

We have clarified this in the new section ‘Recruitment and data collection’.

How was initial guidance provided? Email?

Initial guidance was provided by email (line 166).

Virtual or in-person meetings?
We have included this in line 192.

How long was the sign-up and matching process?
We have added this from line 182.

How were mentors identified?
We have added this from line 156.

*Results*
There are several universities identified as being a part of AIMMS were all of these institutions represented in the evaluation? Did they all have participants?

Participants were not asked to identify themselves in either questionnaire. We have clarified this in lines 236-239. We do not know which mentors and mentees answered the questionnaires or where they worked.

What information is being shared in the grey shaded box (lines 208-210).
These are quotations from mentees/mentors in response to the question “Do you feel that having a successful career in academic medicine has been more challenging because you are a woman from an ethnic minority?” We have clarified this in lines in each of the boxes.

What does sharing the age and ethnicity for the follow up questionnaire provide? Were different participants surveyed?

All participants were asked to complete both the baseline and follow-up questionnaires, but unfortunately not everybody did. The surveys are anonymous so we were not able to track participants’ responses between the two time points. The purpose of including the age and ethnicity profiles for both the baseline and follow-up was to be transparent about the demography of the respondents at both timepoints.

Unclear what Figures are presented in the text.

There are titles for Figures 1, 2, and 3, but there are not any figures connected with the titles.

The figures are uploaded as separate documents as per journal guidelines.

There are grey shaded boxes spread throughout the results without labels/titles.

Thank you. We have corrected this throughout by providing references to the boxed-out quotations in the text. The quotations were sourced from free text responses and selected to illustrate findings. We have clarified this at line 258.

Lines 268-269 Were participants eligible to be both mentors and mentees?

Yes, participants could choose to be both. We have clarified this in line 172.

What was the point in surveying participants prior to and after 6 months if no comparison of the data gathered is presented or discussed in the manuscript?
In the baseline questionnaire we wanted to find out what people’s expectations were of the scheme before they started mentoring. We also wanted to find out if they thought there were challenges associated with being a woman from an ethnic minority background. The follow-up questionnaire aimed to evaluate if expectations had been met and if so, in what way. We expand on this point in the next paragraph.

*Discussion*
There is no discussion of the information from the pre-survey. What did that information provide to the evaluation? Were mentees/mentors expectations met? Overall, more discussion of the results is needed.

Due to the limited wordcount, we did not dedicate much discussion to this comparison, mentioning it in the first two paragraphs where we say what participants’ expectations were and then comment on how they were met by the scheme. We have expanded on this from line 407 and added some discussion about Table 1 from line 398.

**Reviewer 2 comments**

Dear Authors,
I congratulate the authors for the wonderful initiative to promote gender equality in Medical and health sciences through mentoring aiming to highlight the significant hidden gaps in current environment in a developed country.

You conclude that mentoring between women from similar backgrounds is valuable and can assist with personal, professional and career development. I would agree with this statement but feel that this should change in the near future. Race, Gender or geographic background should be no longer an impediment but the should help develop a better scientific and global harmony among the scientific community leading to innovation, advancement and 'out of the box' thinking & mentoring advances.

Thank you very much for your comments and we fully support your hope that one day methods to help career progression will no longer be needed for women from ethnic minority backgrounds.

This is a well designed and executed study to bring an important aspect of mentoring for women from ethnic minority backgrounds working in academic medicine and health sciences. You have acknowledged that this is a small number of 28 participants study with a scope for multi centric collaborative global initiative.

You explore various aspects of mentoring via formative and summative evaluation concluding that the AIMMS scheme promotes positive model for equitable environment and opportunity for women in health sciences.

This will open up multiple corridors for women empowerment and medical research. We are not too far from this concept to become a global initiative breaking all the barriers. I believe this study is a powerful step forward.

Can you make amendments:
1. Please add a paragraph before conclusion on Limitations of the study.
Thank you, we have added a heading on line 435 to highlight limitations.

2. Please add a note on how this can be expanded to LMIC and across various medical and allied health faculties globally?
In the last line of discussion we have mentioned the scheme could be applied globally.

Thank you.

Best wishes.

Thank you very much for your considered feedback. We hope we have been able to take account of all your comments in our revisions.

**Reviewer 3 comments**

visual representation of results shall be done. data illustration shall be improved.

Thank you. Visual representation of some of the results/data can be found in Figures 1 to 3 which are separate files.

proper criteria for objective development shall be used. evaluation is not mentioned under the objective and aims section.

The objectives included in the introduction are the objectives for the mentoring scheme rather than the evaluation.

what was the evaluation strategy, how was it measured?

We have added that the AIMMS Founding Committee designed the strategy to include both formative and summative elements (line 160).

Thank you very much for your considered feedback. We hope we have been able to take account of all your comments in our revisions.

**Reviewer 4 comments**

The evaluation of the AIMMS Mentoring scheme provides valuable insights into the benefits of tailored mentoring for women from ethnic minority backgrounds in academic medicine and health sciences. While the study highlights significant positive outcomes, addressing the methodological limitations and expanding the scope of future research will enhance the understanding and impact of such initiatives.

Thank you. We have now highlighted and expanded the limitations subsection within the Discussion section.

With only 16 pairs (ten mentees and four mentors completing the follow-up), the sample size is relatively small, limiting the generalizability of the findings. The low number of participants who completed the follow-up questionnaire (14 out of 32) may introduce response bias, as those who did not respond could have different experiences. The study lacks Control Group: The absence of a control group makes it difficult to attribute observed outcomes solely to the mentoring scheme, as other factors might have influenced the results.

We agree we kept the analysis simple but this fulfilled the aim of the evaluation. We agree a larger evaluation of similar schemes is desirable if resources allow. We reported all the data in tables other than the free text so that readers could be fully informed. We did not undertake any detailed statistical comparisons due to the small sample size

There is limited specificity: The findings are broadly positive but lack detailed statistical analysis or specific metrics, making it difficult to quantify the extent of development or satisfaction. The overwhelmingly positive feedback may overlook potential negative aspects or challenges that were not fully explored or reported. Providing more specific data and employing statistical methods would offer clearer insights into the scheme's effectiveness.

The study focuses on short-term outcomes, with limited insight into the long-term impact and sustainability of the mentoring relationships and their effects on career progression.
Conducting follow-up studies beyond six months would help assess the long-term impact and sustainability of the mentoring relationships.

We also agree we were only looking at short-term impacts. This was because resources were limited. We have added this point to the new ‘limitations’ section (from line 434). We have also added a point about future research (from line 443).

Thank you very much for your considered feedback. We hope we have been able to take account of all your comments in our revisions.
